# Super Natural II—a database of natural products

**DOI:** 10.1093/nar/gku886

**Published:** 2014-10-09

**Authors:** Priyanka Banerjee, Jevgeni Erehman, Björn-Oliver Gohlke, Thomas Wilhelm, Robert Preissner, Mathias Dunkel

**Affiliations:** 1 Structural Bioinformatics Group, Charite-University Medicine Berlin, Institute of Physiology, Lindenberger Weg 80, 13125 Berlin, Germany; 2 Graduate School of Computational Systems Biology, Humboldt-Universität zu Berlin Invalidenstrasse 42, 10115 Berlin, Germany; 3 German Cancer Consortium (DKTK), Im Neuenheimer Feld 280, 69120 Heidelberg, Germany; 4 Institute of Food Research, Norwich Research Park, Colney, Norwich, UK

## Abstract

Natural products play a significant role in drug discovery and development. Many topological pharmacophore patterns are common between natural products and commercial drugs. A better understanding of the specific physicochemical and structural features of natural products is important for corresponding drug development. Several encyclopedias of natural compounds have been composed, but the information remains scattered or not freely available. The first version of the Supernatural database containing ∼50 000 compounds was published in 2006 to face these challenges. Here we present a new, updated and expanded version of natural product database, Super Natural II (http://bioinformatics.charite.de/supernatural), comprising ∼326 000 molecules. It provides all corresponding 2D structures, the most important structural and physicochemical properties, the predicted toxicity class for ∼170 000 compounds and the vendor information for the vast majority of compounds. The new version allows a template-based search for similar compounds as well as a search for compound names, vendors, specific physical properties or any substructures. Super Natural II also provides information about the pathways associated with synthesis and degradation of the natural products, as well as their mechanism of action with respect to structurally similar drugs and their target proteins.

## INTRODUCTION

Natural products (NPs) are classical starting points for drug discovery. All herbal-based medicines are derived from natural compounds (1). NPs were involved in the development of ∼64% of all drugs (2). For instance, easily available drugs such as lovastatin, paclitaxel, penicillin and silibinin were either directly or indirectly derived from NPs. NPs are an invaluable source of inspiration for organic chemists to synthesize novel drug candidates (3–6). Macrocycles, ring structures with more than 12 atoms, are a typical structural feature of natural compounds. They help to organize the overall molecule structure such that key functional groups can specifically interact with binding sites of target molecules such as proteins, resulting in few or no entropic loss on binding. Often macrocyclic NPs such as erythromycin, rapamycin, tacrolimus possess favorable drug-like physicochemical and pharmacokinetic properties like lipophilicity, metabolic stability, increased solubility and bioavailability (7). Such properties are important for protein–protein interaction targets (8). It is known that more NP-like molecules are needed for corresponding library design of the pharmaceutical industry (9,10). An interesting example for NP-based drugs starts with camptothecin isolated from the bark and stem of *Camptotheca acuminata* (11), which inhibits the DNA enzyme topoisomerase I. Because of low solubility and high adverse drug reaction during clinical trials (12), it was modified to the analogs topotecan and irinitecan (13). These analogs have been approved for cancer chemotherapy. The availability of NP databases is important for *in silico* screening in drug discovery. However, NP databases, such as the Dictionary of Natural Products (http://dnp.chemnetbase.com) and Natural Product Alert (14), are often commercial or freely available only with restricted information.

Better knowledge of the anabolism and catabolism of NPs leads to a relevant understanding of their ecological role. Often, primary metabolic pathways are single product and target-oriented (like tryptophan is synthesized in the tryptophan pathway, cholesterol is synthesized in the cholesterol pathway), whereas the pathways of the secondary metabolism are diversity-oriented, resulting in more than one product (15). This unique chemical diversity of secondary metabolites is one of the reasons for the continuing scientific interest in NPs (16,17,18). Nature has designed and selected secondary metabolites for different purposes. They often act as ‘defense compounds’ and were optimized during evolution for specific interactions with biological receptors (15,16).

Some NPs are toxic and produce adverse effects on cells or the whole organism. For instance, consumption of alpha-amanitin, a toxic peptide produced by *Amanita* mushrooms (19), can lead to irreversible kidney and liver damage (20). This NP molecule is considered as one of the deadliest compounds known.

Super Natural II is a freely available, web-based and easy to access database of NPs. Super Natural II contains a lot of information for each compound, including NP class, predicted toxicity class, mechanism of action (MoA) and pathways information. Super Natural II provides possible toxicity alert for the use of a particular natural compound. However, the absence of such toxicity prediction or alert for a compound should not be taken as an indication of safety. The toxicity prediction for the database compounds is based on their structural similarity to the known toxic compounds, calculated using ProTox (21). ProTox shows good performance in comparison to other commercial and free toxicity prediction methods.

To the best of our knowledge, Super Natural II is the first publicly available database of natural compounds with as many as 326 000 molecules and the corresponding wealth of additional information. Super Natural II provides information on MoA, pathways information and toxicity information integrated into one single platform. A comparison of Super Natural II to other NP databases is provided in Supplementary Table S1.

## MATERIALS AND METHODS

The Super Natural II database contains ∼326 000 unique compounds, collected from 16 suppliers (as listed on the Super Natural II website) and five freely available databases (22–26).

### Data preparation

The collected raw data from 21 sources were standardized using the chemoinformatic pipeline of the Konstanz Information Miner (KNIME) (27) (Steps 2–5) and ChemAxon (http://www.chemaxon.com). (Step 1): (1) standardizing files into the structure-data (SD) format, using JChem. In this process, incomplete structures were deleted, charges normalized and ions and smaller parts (e.g. water, salts) removed. (2) Reading standardized SD files, (3) generating InChIKeys (RDKit to InChi), (4) removing duplicates and (5) writing new SD files (see Supplementary Figure S3). Corresponding supplier information is mapped to each unique structure. The compounds are listed in the final database with a unique identifier (SN_ID). The 2D structure of each compound provided by the suppliers was used to generate 3D structures (Discovery studio, Accelrys Inc., http://www.accelrys.com/dstudio). A set of physicochemical descriptors such as molecular weight, log*P*, H-bond donors, H-bond acceptors, number of rings, aromatic rings, number of bonds and the number of heavy atoms was computed for each compound using JChem 6.1.3 (November 2013), ChemAxon (http://www.chemaxon.com).

### Similarity search and substructure search

The structural similarity search was implemented using Open Babel (28). The Tanimoto coefficient (29) is used to measure the 2D similarity. It compares the structural similarity between the query molecule and the database entries using a concatenated fingerprint of the ‘FP2’ and ‘FP4’ Open Babel fingerprints calculated by Mychem (http://mychem.sourceforge.net). The Tanimoto coefficient is a number between 0 and 1 (1 corresponding to ‘maximum similarity’) (30). Pre-calculated fingerprints for all database entries are stored as blob objects in the MySQL-database. For a query structure it is calculated during the search. The top 15 results are returned.

For the substructure search, a fast-search index for all database compounds was created using Open Babel (28). The computed index is then queried by Simplified Molecular Input Line Entry Specification (SMILES) (31); resulting in up to 4000 hits per substructure search.

### Classification

Due to the greater structural diversity of NPs compared to synthetic compounds, an increased therapeutic spectrum can be covered (32). Different classification schemes for NPs exist; Super Natural II has implemented a classification by structural characteristics such as alkaloid, amino acid or fatty acid.

### Toxicity prediction

The toxicity class for each compound in the database was calculated using ProTox (21). The toxicity is categorized into six classes (ranging from classes I to VI) based on the lethal dose (LD50) values in mg/kg body weight in rodents. The toxicity class is predicted for ∼170 000 compounds; for unpredicted compounds (out of prediction range) the class is displayed as zero. For a better understanding of the toxicity prediction a link to the ProTox web server is provided. The number of NPs predicted for each toxicity class is reported in Supplementary Table S2.

### Mechanism of action

Drug–target relations were taken from SuperTarget (33), which comprises >195 000 pharmacologically active compounds for which interactions are quantitatively known. These drugs were compared to all the Super Natural II compounds using a Tanimoto coefficient of 0.8 and above; the corresponding information was stored. Each NP was then mapped to its predicted target using the information from the drug-NP similarity mapping. It is generally accepted that structurally similar compounds have usually similar biological properties (34). The target prediction was computed using SuperPred web server (35), which has a prediction accuracy of 75.1%. Not all the NPs are yet assigned to a target, so similarity of a query NP to a ligand with known receptor association(s) may provide useful insight. For instance, search with target ‘Bcl-2’ and ‘Apoptosis regulator Bcl-2’ results in a list of drugs (15 hits) that are acting on this specific target. This drug could be a natural compound like ‘Melatonin’ (36); additionally, with the help of pathway information on the results page, more specific facts on the pathways involved (such as Apoptosis, Alzheimer's disease) and corresponding species information can also be obtained.

### Pathways

Considering information from KEGG (22) pathways often helps to better understand a specific NP–target protein interaction. In order to display pathway maps, NPs were mapped to potential targets with respect to their similarity to the reference drugs (see ‘mechanism of action’ section).

For instance, given a search for the organism ‘Homo sapiens’ and the pathway ‘Apoptosis’, the result page will display the corresponding pathway map with all known targets highlighted. A cursor-over on the target will display drugs acting on the target (up to 15 hits), together with similar NPs and related similarity score. Currently, the database is featuring all Homo sapiens pathways, as well as pathways from bacteria and fungi.

### Clustering

A compound clustering based on structural similarity using Tanimoto coefficients was performed for the entire data set. The clustering was generated using an algorithm based on DBSCAN (density-based spatial clustering of application with noise) (37). The structural similarity clustering was calculated using similarity measures based on the concatenated Open Babel FP24 fingerprint, calculated using Mychem (http://mychem.sourceforge.net). The clustering was based on the corresponding similarity matrix with Tanimoto coefficient entries of 0.8 and above. The clustering is an effective tool to compare structural similarities between a large set of natural compounds. It results in ∼27 000 clusters with a minimum size of four compounds. Larger clusters are broken down into smaller subsets, containing at most 30 compounds in order to simplify the interpretation of the complex clustering results. The clustering results are visualized in an interactive heat map where red color corresponds to high similarity and light colors to low. A cursor-over on the heat map helps to view the corresponding compound structures.

### Server, database and system requirements

Super Natural II is based on a relational MySQL database (http://www.mysql.com). To handle the chemical information within the database, the MyChem package (http://mychem.sourceforge.net/) is used. For most of its functions, MyChem relies on the Open Babel toolbox (http://openbabel.org). The Super Natural II website is built using PHP (http://www.php.net/), JavaScript (http://www.java.com/), Ajax; web access is enabled via an Apache HTTP Server (http://http.apache.org/). The site is best viewed in Mozilla Firefox and also functional on alternate browsers like Google Chrome and Microsoft Internet Explorer.

## SEARCH OPTIONS

**Figure 1.**
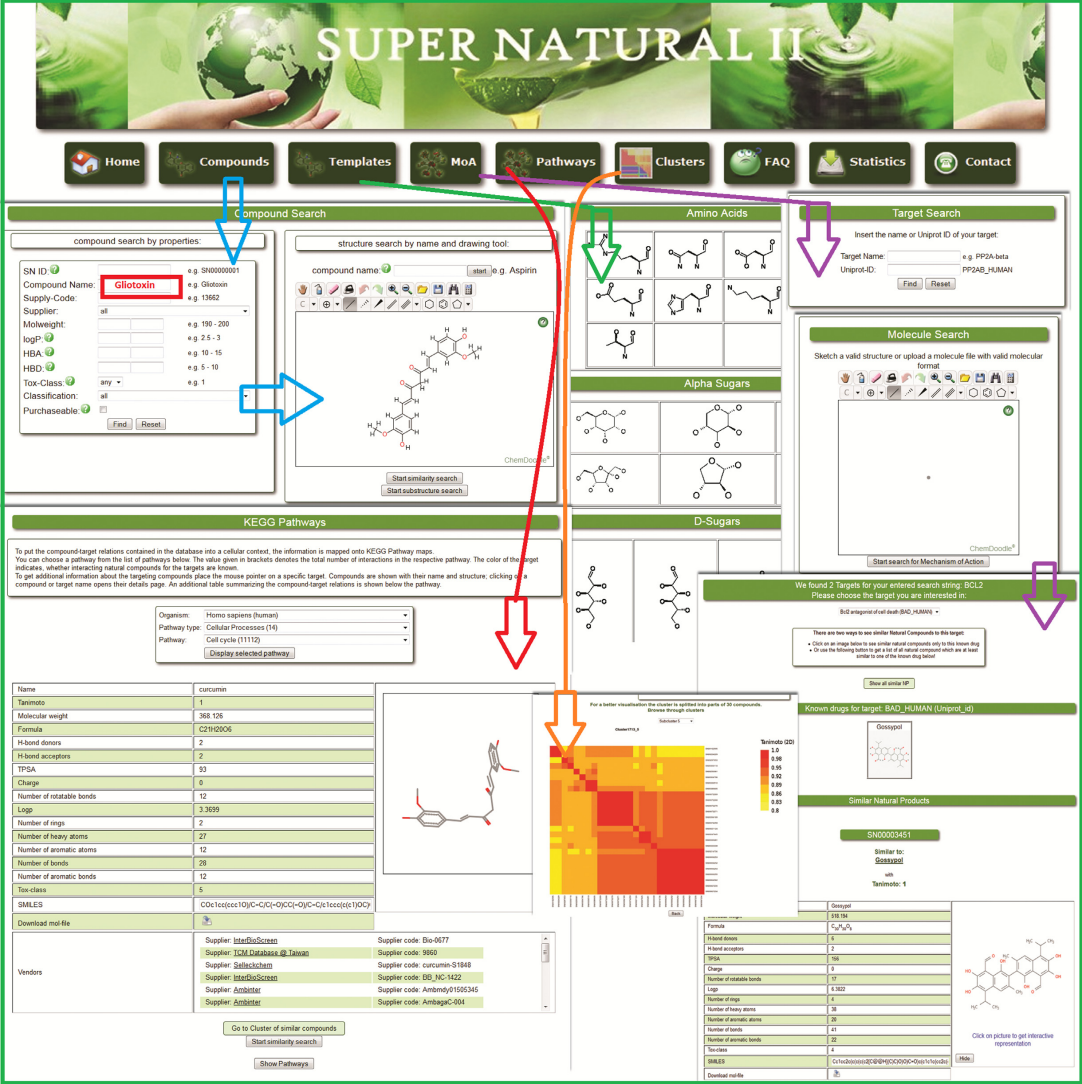
Super Natural II database search options. The compound search (blue arrow) enable search via compound name, supplier, molecular weight, log*P*, hydrogen-bond donor and acceptor, classifications, toxicity class as well as compounds that are purchasable. Template search (green arrow) allows searching via a pre-defined template. MoA search (purple arrow) can be performed for a molecule structure or a target name; clusters (orange arrow) can be searched via name, SNID (unique identifier for the Super Natural II database), IUPAC name or SMILES. All search results contain information about physicochemical properties, toxicity class, vendor and pathway. Cluster results are displayed using heat maps and related similarity values for compounds of the cluster. Pathway information (red arrow) can be obtained for specific species and pathway. Additionally, the molecule structure is shown.

Natural compounds can be found in the Super Natural II database via several search methods. A web-based search tool is available, incorporating a molecular drawing interface. Users can also search for compounds by properties, chemical structures or by a combination of criteria. Additionally, the integrated PubChem (38) search for a compound name is implemented. The search results page shows the main properties and the chemical structure of the compound, as well as a link to download the structure file in the Mol2 format. The web system was designed to allow a rapid and detailed response to specific queries. Important search options are as follows (Figure 1):

- First, a compound name or known supplier code can be used to retrieve information from the database. To get a subset of molecules having particular physicochemical properties, a search can be refined with more options like molecular weight, log*P* value, hydrogen-bond acceptor, hydrogen-bond donor and classification. Additionally, one can also look for toxic natural compounds using the toxicity class filter.
- Using the ChemDoodle (http://www.chemdoodle.com) sketcher, a user can build or import a molecular structure and perform a similarity or substructure search with compounds of the Super Natural II database.
- Substructure search can also be done by pre-defined scaffolds via template search. Here, users can look for compounds in the database containing specific groups like certain amino acids, alpha sugars, beta sugars, D-sugars, aromatic rings, bicyclical rings, fused rings, heterocyclic or polycyclic rings, etc.
- An MoA search enables users to screen NPs for information on their putative molecular targets and corresponding pathways. Similarly, the user can specify a target protein as search term (Uniprot ID/protein name) and get information on natural compounds which are predicted to interact with the specified target protein (see 'mechanism of action' section).
- The pathway search enables users to display known NPs and interacting protein targets associated with any pathway map from KEGG. The pathways can be searched by species, pathway type and function. Each protein target interacting with an NP is highlighted in yellow. A cursor-over option shows detailed information on similar compounds.

## CONCLUSION

The Super Natural II database is a freely available resource with different embedded search functions. It allows a simple access to the growing number of available natural compounds. The compounds of the database comprise a rich chemical diversity and a wide spectrum of biological and pharmacological activities. Associated pathways and known and predicted mechanisms of action supply additional information. We expect that Super Natural II will be useful for studies involving virtual screening, metabolomics and the design of new compounds. The information provided by Super Natural II about structures and physicochemical properties of ∼326 000 NPs will contribute to future drug development.

## AVAILABILITY

Super Natural II is available through the website without registration: http://bioinformatics.charite.de/supernatural.

## SUPPLEMENTARY DATA

Supplementary Data are available at NAR Online.
